# Supplementary material for: Epidemiology, clinical features, and impact of food habits on the risk of hepatocellular carcinoma: A case-control study in Bangladesh
Source: PLoS One. 2020 Apr 27;15(4):e0232121. doi: 10.1371/journal.pone.0232121 (PMC7185601; doi:10.1371/journal.pone.0232121)
Supplement: S1 Table — (DOCX) [file pone.0232121.s001.docx]

Supplementary

Table S1. Bivariate association between food habit and hepatitis B or C positive against the others.

| Risk factor | Levels | B/C Positive | Other | p |
| --- | --- | --- | --- | --- |
| Tea consumption | No | 12 (26.7) | 6 (17.1) | 0.420 |
|  | Yes | 33 (73.3) | 29 (82.9) |  |
| Tea with condensed milk | No | 20 (44.4) | 13 (37.1) | 0.648 |
|  | Yes | 25 (55.6) | 22 (62.9) |  |
| Tea consumption (bag) | No | 34 (75.6) | 28 (80.0) | 0.789 |
|  | Yes | 11 (24.4) | 7 (20.0) |  |
| Alcohol use | No | 43 (95.6) | 34 (97.1) | 1.000 |
|  | Yes | 2 (4.4) | 1 (2.9) |  |
| Rice intake | High | 43 (95.6) | 31 (88.6) | 0.396 |
|  | Moderate | 2 (4.4) | 4 (11.4) |  |
| Red meat intake | High | 3 (6.7) | 3 (8.6) | 1.000 |
|  | Moderate | 42 (93.3) | 32 (91.4) |  |
| White meat intake | Low | 25 (55.6) | 15 (42.9) | 0.367 |
|  | Moderate | 20 (44.4) | 20 (57.1) |  |
| Egg intake | High | 27 (60.0) | 23 (65.7) | 0.647 |
|  | Moderate | 18 (40.0) | 12 (34.3) |  |
| Wheat intake | Low | 36 (80.0) | 25 (71.4) | 0.433 |
|  | Moderate | 9 (20.0) | 10 (28.6) |  |
| Leafy vegetable intake | Low | 31 (68.9) | 24 (68.6) | 1.000 |
|  | Moderate | 14 (31.1) | 11 (31.4) |  |
| Nonleafy vegetables | Low | 22 (48.9) | 14 (40.0) | 0.500 |
|  | Moderate | 23 (51.1) | 21 (60.0) |  |
| Fruit intake | Low | 45 (100.0) | 30 (85.7) | 0.014 |
|  | Moderate |  | 5 (14.3) |  |
| Fish intake | Low | 35 (77.8) | 27 (77.1) | 1.000 |
|  | Moderate | 10 (22.2) | 8 (22.9) |  |
| Milk intake | Low | 39 (86.7) | 27 (77.1) | 0.375 |
|  | Moderate | 6 (13.3) | 8 (22.9) |  |
| Weight status | Normal | 37 (82.2) | 26 (74.3) | 0.421 |
|  | Overweight | 8 (17.8) | 9 (25.7) |  |
