# Supplementary material for: Epidemiology, clinical features, and impact of food habits on the risk of hepatocellular carcinoma: A case-control study in Bangladesh
Source: PLoS One. 2020 Apr 27;15(4):e0232121. doi: 10.1371/journal.pone.0232121 (PMC7185601; doi:10.1371/journal.pone.0232121)
Supplement: S1 Questionnaire — (PDF) [file pone.0232121.s002.pdf]

Liver Cancer (Hepatocellular Carcinoma) Screening Questionnaires

1. ID NO: [ ]

2. Date: / /

Personal Information

3. Name:

4. What is your date of birth?

[ ] Year [ ] Month [ ] Day

Age:

5. Sex:

6. Height:

7. Weight:

8.Telephone number:

9. What is your educational background?

☐ Illiterate ☐ Primary ☐ Secondary (SSC)

☐ Higher secondary (HSC) ☐ Graduation/ Post graduation

10. What is your current job? Please choose among those in parentheses. If you hold more than one job or change jobs, please check all that apply.

a) Agriculture (Farmer)	b)Fishery (Fisherman)	c)Business/ company worker
d)Office worker	e)Private/ self-employed	f)Professional, e.g., doctor, lawyer, researchers
	g)Housewife	h)Housewife
i)No job	J)Other	

For how many years have you held this job? ☐ years

If you are retired and do not have a job now, please provide your previous job.

☐ years Descriptions: ☐

11. Have you ever had occupational exposure to the following materials? If yes, please check any of the following materials you have been exposed to.

	No
Solvents, dust, lead, undesired noise, vibration, high voltage, ionizing radiation, foul odors, pesticides<br>Other (please specify ________________)	

12. At present, how much you earn per month? / Monthly income of your family. Please choose among those in parentheses.

☐ Less than 5,000/- ☐ 5,001/- to 10,000/- ☐ 10,001/- to 20,000/-

☐ 20,001/- to 50000/- ☐ 50,001/- to 100000/- ☐ >100000/-

13. Present address:

Village	
Post	
Thana	
District	

How many years have you lived at your present address? ☐ years

14. Permanent address:

Village	
Post	
Thana	
District	

How many years have you lived at your permanent address? [ ] years

Family history

15. Marrital status : ☐Married ☐Unmarried

16. How many siblings and children do you have? Do not include yourself.

	Sisters	
Alive		
Deceased		

	Brothers	
Alive		
Deceased		

	Children	
Alive		
Deceased		

17. Have your parents / immediate family had any of the following diseases?

	Father	Mother	Brothers	Sisters	Spouse
Liver cancer					
Any cancer except liver cancer (site)					
Diabetes					
Myocardial infarction					
Hypertension					
Stroke					

Smoking and drinking questions

18. Have you ever been a smoker?

0. No	1. Yes

If yes, how old were you when you began smoking? ☐ Years

Do you currently smoke cigarettes?

0. No	1. Yes	2. Occasionally

If you no longer smoke, how old were you when you quit smoking?

☐ Years

If yes, how many cigarettes a day do you smoke? ☐ Cigarettes/ day

19. Have you ever lived with a smoker for more than 10 years?

0. No	1. Yes

If yes, how old were you whwn you were living with them?

0. Younger than 20 years old	1. Older than 20 years old	2. Both

20.. How often are you exposed to passive smoking (more than 1 hour/day) outside the house, such as at work?

0. Almost never	1. 1-3 days a month	2. 1-4 days a week	3. Almost everyday

21. How often do you drink alcoholic beverages such as beer, whiskey, rum, brandy, or wine?

0. Almost never	1. 1-3 days a month	2. 1-2 days a week	3. 3-4 days a week	4. 5-6 days a week	5. Everyday

22. If you drink 1-2 days /week, then how much of the following do you drink every day?

Beer (ml)	No. of cans _
Whiskey	How much ml _
Ram	How much ml _
Brandy	How much ml _
Wine	How much ml _
Others	How much ml _

23. Do you smoke more cigarettes than usual while drinking alcohol?

0. Do not smoke while drinking	2. Smoke as usual	3. More than usual

24. Does your heart beat faster than usual or do you get a headache soon after drinking alcohol?

1. Yes	2. Probably yes	3. No change	4. Don’t know

Baseline dietary questions

25. How much water do you drink in a day? [ ] litre

26. Do you drink boiled water?

0. No	1. Yes

27. Do you drink water from unsafe sources such as tea stall, hotels etc.?

0. No	1. Yes

28. How many days per week do you eat the following meals? Indicate the frequency with circle.

	Seldom	1-2 days/week	3-4 days /week	All most every day
Breakfast	0	1	2	3
Lunch	0	1	2	3
Dinner at home	0	1	2	3
Snacks between meals	0	1	2	3

29. On average, how many plates (normal size) of rice do you eat per day

0. Less than one plate per day	1. Approximately…………..plate

30. How often do you have pulse soup?

0. Almost never	1. 1-2 days/week	2. 3-4 days/week	3. Almost everyday

If almost every day, how many cups on average do you have a day? [ ] cup/day

31. How do you like the following kinds of food? Please check

Preferences/Dislikes	Like very much	Like a little	Dislike
Food rich in oil	1	2	3
Spicy food	1	2	3
Very salty foods	1	2	3
Sour food	1	2	3
Very sweet foods	1	2	3
Hot food and drinks	1	2	3

32. How often do you have fried foods such as stir-fry or deep fry?

0. Almost never	1. 1-2 days/week	2. 3-4 days/week	3. Almost daily

33. Do you avoid the burned/charred part of fish or meat?

0. No	1. Yes

34. What kind of cooking method do you use most often when you cook the following foods?

	Boiled	Grill	Deep-fry	Stir-fry	Others
Meats	1	2	3	4	5
Fish	1	2	3	4	5
Vegetables	1	2	3	4	5

35. Write the frequency with which you eat (or do not eat) each of the following foods.

Frequency	Never	Times/ month	Times /week	Times/ day	Gram/ servings
Rice					
Noodles(not instant)					
Roti					
Bread					
Butter or margarine					
Seasonal fruits (litchies, mangoes, jackfruits, blackberries, dates, guavas, pineapple, others……………………)					
Non-seasonal fruits (papayas, bananas, coconuts, apples, grapes, oranges, others………….)					
Leafy vegetables (water spinach, pumpkin leaves, taro stem, indian spinach, spinach, red amaranth cauliflower, cabbage)					
Non-leafy vegetables, green (lady's finger,					

tomato, balsam apple, others……………………)					
Non-leafy vegetables, colorful (brinjal, carrot, pumpkin, others………...)					
Others vegetables (bean, bean seed, cowpea, peas……………………..)					
Potato, sweet potato					
Pulse					
Mayonnaise					
Mushroom					
Egg (number)					
Milk products					
Cheese					
Red meat					
White meat					
Liver					
Freshwater fish					
Cultivated fish (anabas, shrimp, carp, tarpon, halibut, barbel, catfish, salmon, troul, flat fish….)					
Sea fish					
Dried and salted fish					
Nut					
Sweet					
Betel leaf, dried scented tobacco, lime, areca nut					
Burger					
Pizza					
Sandwich					
Chicken Fry					

36. Are you careful about your salt intake?

0. No	1. Yes

How much salt you intake?

0. High	1. Low	2. Moderate

37. Are you careful about your cholesterol intake?

0. No	1. Yes

38. Do you eat a lot of green and yellow vegetables?

0. No	1. Yes

39. Are you careful about fat intake?

0. No	1. Yes

40. Compared to average, how would you describe the volume of food you typically eat in a meal?

0. Much less	1. Less	2. Same	3. More	4. Much more

41. How do you describe your usual meal?

0. Eat until half-full	2. Eat until 80% full	3. Eat until full

42. Circle the frequency of your average consumption of each of the following beverages:

Frequency		Almost Never	Times/ month	Times/ week	Times / day	ml / servings
Tea (cup)						
Tea with tea bag (cup)	Outside home					
	Inside home					
Tea with condensed milk (cup)	Outside home					
	Inside home					
Coffee (cup)						
Milk (glass)						
Soft drinks (bottle)						
Fruit juice (glass)						
Vegetable juice (glass)						

43. How many teaspoons of sugar do you use per cup of coffee or black tea?

(If do not take sugar, please indicate “0”) [ ] teaspoons

44. How often do you take painkiller?

0. Never	1. Seldom	2. 1-4 days per week	3. Almost everyday

45. How often do you take oral contraceptive pill? (For women only)

0. Never	1. Seldom	2. 1-4 days per week	3. Almost everyday

46. How often do you participate in sports or physical exercise?

0. almost never	2. 1-3days per month	3. 1-2 days per week	4. 3-4 days per week	5. Almost everyday

47. How many hours do you sleep usually daily? [ ] hours

Complains with duration /First clinical symptoms of patients

48. Please check, If you have those following symptoms:

Complains	0. No	1. Yes	If yes then the duration
Right hypochondriac / Epigastric pain			
Abdominal pain			
Weight loss			
Weakness			
Abdominal swell			

Yellow eye and skin			
Fever			
Headache			
Anorexia			
Vomit feeling			
Yellow spot in the body			
Pain in the right shoulder			
Fatigue			
Itching			
Unconsciousness			
Tendency to bleed more easily			
Others…………			

Past medical history

49. Do you ever affected with HBV? ☐1. Yes ☐0. No

If yes, then from when had you affected? ☐

Did you take any treatment after you had infected? ☐1. Yes ☐ 0. No

Name of the drug: Dose:

Any interruption of treatment: ☐ 1. Yes ☐ 0. No

If yes, then from when did it interrupt? ☐

50. Do you ever affected with HCV? 1. Yes 0. No

If yes, then from when had you affected? ☐

Did you take any treatment after you had infected? ☐ 1. Yes ☐ 0. No

Name of the drug: Dose:

Any interruption of treatment: ☐ 1. Yes ☐ 0. No

If yes, then from when did it interrupt? ☐

51. Have you ever diagnosed with any of the following disease? Please check the following:

Name of the disease	0. No	1. Yes	If yes, then duration
Liver cirrhosis			
Hemochromatosis			
NAFLD(Non-alcoholic Fatty Liver Disease			
Diabetes			
Stroke			
Hypertension			
Myocardial infection			
Asthma			
Chronic bronchitis			
Allergy			
Kidney disease			
Peptic gastric			
Biliary stone			
Others…………..			

52. Do you have any cancer other than liver cancer?

0. Yes	1. No

53. Did you have any kind of operation before?

If yes then what types of operation:

- ☐ General operation
- ☐ Cesarean operation
- ☐ Dental operation
- ☐ Transplantation

54. Have you ever received blood?

0. Yes	1. No

55. Have you ever use a used syringe?

0. Yes	1. No

56. Patients BMI = weight (kg) / height (m^2)

- ☐ Normal weight (BMI 18.5-24.99)
- ☐ Overweight (BMI 25-29.99)
- ☐ Obesity 1st stage (BMI 30-34)
- ☐ Obesity 2nd stage (BMI 35-39.99)
- ☐ Obesity 3rd stage (BMI 40+)

Obesity?

0. Yes	1. No

Information related to liver cancer

57. Type of liver cancer have you affected? ☐ Primary ☐ Secondary

58. When did your liver cancer first diagnose? [ ]

59. Have you ever heard about liver cancer?

0. Yes	1. No

60. Stage of liver cancer according to BCLC:

Stage A
Stage B
Stage C
Stage D

61. From when have you started treatment? [ ]

62. In which stage the cancer was diagnosed? [ ]

63. What type of treatment have you received?

Surgery	Chemoembolization	Systemic therapy	Ablation	Others…..

64. What type of diagnosis have you done for the detection of cancer?

CT scan	MRI	Ultra sound	Biopsy	Biochemical test	Others….

Tumor markers

65. AFP (ng/ml) : ☐ <200 ☐ 200-1000 ☐ >1000

66. CEA (ng/ml) :

67. Endoscopy of upper GIT :

68. The diameter of the tumor:

Number of tumors:

Physical examination

69. Anemia: ☐ Present ☐ Absent; If present, ☐ Mild ☐ Moderate ☐ Severe

70. Jaundice: ☐ Present ☐ Absent; If present, ☐ Mild ☐ Moderate ☐ Severe

71. Temperature (^{0}F) :

72.Respiratory rate (/min):

73.Pulse(/min):

74.Systolic BP (mm of Hg):

75.Diastolic BP (mm of Hg):

76.Hepatomegaly: ☐ Present ☐ Absent

77.Hepatic bruit:

78. Ascites: ☐ Present ☐ Absent; If present, ☐ Grade-1 ☐ Grade-2 ☐ Grade-3

79. Splenomegaly: ☐ Present ☐ Absent

80.Cachexia/ muscle wasting: ☐ Present ☐ Absent

81. Stigmata of CLD:

- ☐ Leuconychia
- ☐ Palmar erythema
- ☐ Spider angioma
- ☐ Gynaecomastia
- ☐ Testicular atrophy

82.Other systemic examination:

i) CVS:

ii) Respiratory:

iii) Nervous system:

iv) Locomotor:

Investigation reports

83. Hb% (g/dl) :
84. TC of WBC (....x 10^9):
85. Differential count- Neutrophil (%):
86. Lymphocyte (%):
87. Monocyte (%):
88. Eosinophil (%):
89. Basophil (%):
90. ESR(mm in 1st hour) :
91. Platelet count (......x10^{12}):
92. HBs Ag: ☐ Positive ☐ Negative
93. Anti HCV: ☐ Positive ☐ Negative
94. Anti HBc(T): ☐ Positive ☐ Negative
95. ALT (U/L) :
96. Prothrombin Time(sec):
97. INR:
98. Serum Albumin(gm/dl):
99. Serum Bilirubin(mg/dl):
100. Child-Pugh Score: ☐ Stage -A ☐ Stage-B ☐ Stage-C
101. Urine R/M/E :
102. Blood Glucose(RBS)(mmol/l) :
103. Serum Creatinine(mg/dl):

Imaging

104. Chest X-ray P/A view :

105. USG of the whole abdomen :

106. Triphasic CT/MRI :

Number of sol: 1 ☐ 2-3☐ >3☐

Size(cm) : ☐ <2 ☐ >2

Hepatic Segments involved:

Vascularity: ☐ Hypervascular ☐ Hypovascular

Portal vein invasion: ☐Yes ☐ No

Extrahepatic metastasis: ☐Yes ☐ No

107. **Cytopathology:**

108. **Medicine prescribed:**

Signature of Data Collector
