# Supplementary material for: Epidemiology, clinical features, and impact of food habits on the risk of hepatocellular carcinoma: A case-control study in Bangladesh
Source: PLoS One. 2020 Apr 27;15(4):e0232121. doi: 10.1371/journal.pone.0232121 (PMC7185601; doi:10.1371/journal.pone.0232121)
Supplement: S2 Questionnaire — (PDF) [file pone.0232121.s003.pdf]

যকৃত ক্যান্সার (হেপাটোসেলুলার কার্সিনোমা) স্ক্রিনিংয়ের প্রশ্নাবলী

[Liver Cancer (Hepatocellular Carcinoma) Screening Questionnaires]

১। আইডি নং ☐

২। তারিখঃ / /

ব্যক্তিগত তথ্যাদি

৩। নামঃ

৪ । আপনার জন্ম তারিখ কবে?

☐ বছর ☐ মাস ☐ দিন

বয়স:

৫। লিঙ্গঃ

৬। উচ্চতাঃ

৭। ওজনঃ

৮। ফোন নম্বরঃ

৯। শিক্ষাগত যোগ্যতাঃ ☐অশিক্ষিত ☐প্রাথমিক শিক্ষা ☐এস এস সি ☐এইচ এস সি ☐স্নাতক ও তদূর্ধ্ব

১০। আপনার বর্তমান পেশা কি? প্রথম বন্ধনী মধ্যে তাদের চয়ন করুন। যদি আপনি একাধিক চাকুরী করে থাকেন বা চাকরি পরিবর্তন করেন তবে দয়া করে প্রযোজ্য সমস্ত কিছুর চেক করুনঃ

ক) কৃষিক্ষেত্র (কৃষক)	খ) মৎস্য (জেলে)	গ) ব্যবসা / সংস্থার কর্মী
ঘ) অফিস কর্মী	ঙ) বেসরকারী / স্ব-কর্মসংস্থান	চ) পেশাদার, উদাঃ, ডাক্তার,
আইনজীবী, গবেষক	ছ) গৃহিনী	জ) চাকরী নেই
ঝ) অন্যান্য...।		

আপনি এই কাজটি কত বছর ধরে করছেন? ☐ বছর

আপনি যদি অবসরপ্রাপ্ত হন এবং এখনই কোন চাকরি না করেন তবে দয়া করে আপনার আগের কাজের তথ্য সরবরাহ করুনঃ

. ☐ বছর বর্ণনাঃ ☐

১১। কর্মক্ষেত্রে নিম্নলিখিত কোন বস্তুর সংস্পর্শে ছিলেন কিনা? হ্যাঁ হলে, তবে দয়া করে নীচের কোন উপাদানের সম্মুখীন হয়েছেন বলে মনে করেনঃ

	না
দ্রাবক, ধূলিকণা, সীসা, অনাকাঙ্ক্ষিত শব্দ, কম্পন, উচ্চ ভোল্টেজ, আয়নাইজিং বিকিরণ, দূর্গন্ধ, কীটনাশক।<br>অন্যান্য (উল্লেখ করুন)..।	

১২। বর্তমানে, আপনি প্রতি মাসে কত আয় করেন? / আপনার পরিবারের মাসিক আয় কত?

☐ ৫,০০০/-এর কম ☐ ৫,০০১/- থেকে ১০,০০০/- ☐ ১০,০০১/- থেকে ২০,০০০/-

☐ ২০,০০১/- থেকে ৫০০০০/- ☐ ৫০,০০১/- থেকে ১০০০০০/- ☐ >১০০০০০/-

১৩। বর্তমান ঠিকানা:

গ্রাম	
পোস্ট	
থানা	
জেলা	

আপনার বর্তমান ঠিকানায় আপনি কত বছর বাস করেছেন? ☐ বছর

১৪। স্থায়ী ঠিকানা:

গ্রাম	
পোস্ট	
থানা	
জেলা	

আপনার স্থায়ী ঠিকানায় আপনি কত বছর বাস করেছেন? ☐ বছর

পারিবারিক তথ্যাদি

১৫। বৈবাহিক অবস্থা: ☐ বিবাহিত ☐ অবিবাহিত

১৬। আপনার কত ভাইবোন এবং সন্তান রয়েছে? নিজেকে অন্তর্ভুক্ত করবেন না

বোন		
জীবিত		
মৃত		

ভাই		
জীবিত		
মৃত		

সন্তান		
জীবিত		
মৃত		

১৭। আপনার পিতামাতার / নিকটাত্মীয় পরিবারে নিম্নলিখিত রোগগুলির কোনটি ছিল?

	বাবা	মা	ভাই	বোন	স্ত্রী
লিভার ক্যান্সার					
যকৃতের ক্যান্সার ব্যতীত অন্য কোনও ক্যান্সার (স্থান)					

ডায়াবেটিস					
মায়োকার্ডিয়াল ইনফার্কশন					
উচ্চ রক্তচাপ					
স্ট্রোক					

ধূমপান এবং মদ্যপান সম্পর্কিত প্রশ্ন

১৮। আপনি কি কখনো ধূমপায়ীছিলেন?

০। না	১। হ্যাঁ

যদি হ্যাঁ, আপনি যখন ধূমপান শুরু করেছিলেন তখন আপনার বয়স কত ছিল? ☐ বছর

বর্তমানে আপনি কি ধূমপান করছেন?

০। না	১। হ্যাঁ	২। মাঝেমাঝে

যদি আপনি আর ধূমপান না করেন,

তবে কত বৎসর বয়সে আপনি ধূমপান ছেড়েছেন?

☐ বছর

যদি হ্যাঁ হয় তবে; প্রতিদিন আনুমানিক কতটি সিগারেট গ্রহন করেনঃ ☐ সিগারেট/দিন

১৯। আপনি কি কখনো কোন ধূমপায়ীর সহিত দশ বৎসরের অধিক সময় বসবাস করেছেন?

০। না	১। হ্যাঁ

↓

যদি হ্যাঁ হয়, তবে কত বৎসর বয়স হতে আপনি ধূমপায়ীর সহিত বসবাস শুরু করেছেন?

১। বিশ বৎসরের কম হতে	২। বিশ বৎসরের বেশি হতে	৩। উভয়

২০। আপনি নিজে ধূমপান না করলেও ঘরের বাইরে দিনে কি পরিমান (প্রতিদিন এক ঘণ্টার বেশি সময়) ধূমপায়ীর সংস্পর্শে আসেন?

০।কখনো না	১।মাসে ১-৩দিন	২।সপ্তাহে ১-৪ দিন	৩।প্রায় প্রতিদিন

২১। আপনি কতবার বিয়ার, হুইস্কি, রাম, ব্র্যান্ডি বা ওয়াইন জাতীয় অ্যালকোহলযুক্ত পানীয় পান করেন?

০।কখনো না	১।মাসে ১-৩দিন	২।সপ্তাহে ১-২ দিন	৩।সপ্তাহে ৩-৪ দিন	৩।সপ্তাহে ৫-৬ দিন	৫। প্রায় প্রতিদিন

২২। আপনি যদি, সপ্তাহে ১-২ দিনের বেশি ড্রিংক করেন, তবে গড়ে প্রতিদিন আপনি কি পরিমান ড্রিংক করেন?

বিয়ার	ক্যানের সংখ্যা _
হুইস্কি	কত মিলি _
রাম	কত মিলি _
ব্র্যান্ডি	কত মিলি _
মদ	কত মিলি _
অন্যান্য	কত মিলি _

২৩।আপনি কি ড্রিংক করার সময় অতিরিক্ত ধুমপান করেন?

০। ধূমপান করি না	১। সব সময়ের মত	২। অতিরিক্ত

২৪। ড্রিংক করার পরপর কি আপনার হৃৎপিণ্ডের সঞ্চালন বেড়ে যায় অথবা আপনার কি মাথা ব্যথা শুরু হয়?

১। হ্যাঁ	২। সম্ভবত হ্যাঁ	৩। না	৪। জানি না

খাদ্যাভাস জনিত প্রশ্ন

২৫। দিনে আনুমানিক কত লিটার পানি পান করেনঃ

২৬। ফুটিয়ে পানি পান করেন কি নাঃ

০। না	১। হ্যাঁ

২৭। আপনি চায়ের স্টল, হোটেল ইত্যাদির মতো অনিরাপদ উৎস থেকে জল পান করেন?

০। না	১। হ্যাঁ

২৮। সপ্তাহে কত দিন আপনি নিচে উল্লেখিত খাবার গ্রহন করেন? অনুগ্রহ করে কতবার উল্লেখ করুন।

	মাঝেমাঝে	সপ্তাহে ১-২ দিন	সপ্তাহে ৩-৪ দিন	প্রায় প্রতিদিন
সকালের নাস্তা	০	১	২	৩
দুপুরের খাবার	০	১	২	৩
রাতের খাবার				
খাবারের মাঝে নাস্তা	০	১	২	৩

২৯। গড়ে প্রতিদিন আপনি কি পরিমান (স্বাভাবিক আকার এর কয় বাসন) ভাত খান?

০। প্রতিদিন এক বাসনের	১। প্রায় ________বাসন প্রতিদিন

৩০। আপনি কি প্রতিদিন ডাল খান?

০।কখনো না	১। সপ্তাহে ১-২দিন	২। সপ্তাহে ৩-৪ দিন	৩। প্রায়

যদি প্রতিদিন খান, তবে কি পরিমাণ (কত কাপ) খান? _____কাপ/দিন।

৩১। নিচের কোন খাবারটি আপনার পছন্দের/অপছন্দের?

পছন্দের/অপছন্দের	খুব বেশি পছন্দ	অল্প পছন্দ	অপছন্দ
তৈলাক্ত খাবার	১	২	৩
Spicy খাবার	১	২	৩
খুব লবনাক্ত খাবার	১	২	৩
টক খাবার	১	২	৩
মিষ্টান্ন খাবার	১	২	৩
গরম খাবার এবং কোমল পানীয়	১	২	৩

৩২। আপনি কি প্রায়ই deep-fry or stir-fry খাবার খান?

০।কখনো না	১। সপ্তাহে ১-২দিন	২। সপ্তাহে ৩-৪ দিন	৩। প্রায়

৩৩। আপনি কি মাছ বা মাংসের পোড়া অংশ খান?

০। না	১। হ্যাঁ

৩৪। আপনি কিভাবে খাবার রান্না করেন, অনুগ্রহ করে টিক দিন।

	সিদ্ধ	Grill	Deep-fry	Stir-fry	অন্যান্য
মাংস	১	২	৩	৪	৫
মাছ	১	২	৩	৪	৫
তরিতরকারি	১	২	৩	৪	৫

৩৫। নিচের কোন খাবারটি আপনি বেশি বেশি খান অথবা খান না?

ফ্রিকোয়েন্সি	কখনো না	বার/মাস	বার/সপ্তাহ	বার/দিন	গ্রাম/বার
ভাত					
ন্যুডলস (তাৎক্ষণিক নয়)					
রুটি					
পাউরুটি					
মাখন বা মার্জারিন					
মৌসুমি ফল (লিচু, আম, কাঁঠাল, ব্ল্যাকবেরি, খেজুর, পেয়ারা, আনারস এবং অন্যান্য ....)					
অ-মৌসুমী ফল (পেঁপে, কলা, নারকেল, আপেল, আঙ্গুর, কমলা এবং অন্যান্য ....)					
পত্রবহুল শাকসবজি (জলের পালং শাক, কুমড়ো পাতা, কচু শাক, ভারতীয় পালং শাক, পালং শাক, ফুলকপি, বাঁধাকপি, অন্যান্য ....)					
পাতাহীন শাকসবজি, সবুজ (ঢেঁড়স, টমেটো, করলা, অন্যান্য ....)					
পাতাহীন শাকসবজি, রঙিন (বেগুন, গাজর, কুমড়ো এবং অন্যান্য ....)					
অন্যান্য শাকসবজি (শিম, শিমের বীজ, কাউপি, মটর,.........................)					
আলু, মিষ্টি আলু					
ডাল					
মেয়নেজ					
মাশরুম					
ডিম (সংখ্যা)					
দুগ্ধ পন্য					
পনির					
গরুর মাংস					
মুরগির মাংস					
কলিজা					
স্বাদু পানির (নদীর) মাছ					
চাষের মাছ (কৈ, চিংড়ি, রুই,					

কাতলা, তেলাপিয়া, পাঙ্গাস, শিং, মাগুর, নলা, রুই, মৃগেল, চিতল..)					
সামুদ্রিক মাছ					
শুঁটকি মাছ এবং লবণাক্ত মাছ					
বাদাম (চীনাবাদাম/ কাজুবাদাম)					
মিষ্টি					
পান, জর্দা, চুন, গুল, সুপারি					
বার্গার					
পিজা					
স্যান্ডউইচ					
চিকেন ফ্রাই					

৩৬। আপনি কি লবণ খাওয়ার ব্যাপারে সতর্ক?

০। না	১। হ্যাঁ

লবণ কি পরিমান খান?

০। বেশি	১। কম	২। পরিমাণমত

৩৭। আপনি কি খাদ্যে কোলেস্টেরল এর ব্যাপারে সতর্ক?

০। না	১। হ্যাঁ

৩৮। আপনি কি প্রচুর পরিমাণে সবুজ এবং হলুদ শাকসবজি খান?

০। না	১। হ্যাঁ

৩৯। আপনি কি চর্বি জাতীয় খাবারের ব্যাপারে সতর্ক?

০। না	১। হ্যাঁ

৪০। গড়ে তুলনামুলকভাবে প্রতিবার আপনি কি পরিমাণ খাবার খান?

১।অনেক	২। কম	৩। একই	৪। বেশি	৫। অনেক বেশি

৪১। আপনার প্রতিবার খাবারের ধরণ কি?

১। অর্ধেক পেট ভর্তি	২। ৮০% পর্যন্ত	৩। পূর্ণ পেট ভর্তি

৪২। নিচের কোনটি আপনি নিয়মিত পান করেন?

ফ্রিকোয়েন্সি		কখনো না	বার/মাস	বার/সপ্তাহ	বার/দিন	মিলি/বার
চা (কাপ)						
লিকার চা (কাপ)	বাড়ির বাইরে					
	বাড়ির ভিতরে					
ঘন দুধ চা (কাপ)	বাড়ির বাইরে					
	বাড়ির ভিতরে					
কফি (কাপ)						
দুধ (গ্লাস)						
কোলা, কোমল পানীয় (বোতল)						
১০০% ফলের জুস (গ্লাস)						
শাকসবজির জুস (গ্লাস)						

৪৩। আপনি প্রতি কাপ চা অথবা কফিতে কত চামচ চিনি খান? ☐☐ চামচ

চিনি না খেলে “০” লিখেন।

৪৪। নিয়মিত বা দীর্ঘদিন ধরে ব্যথার ঔষধ সেবন করেন কিনা?

০। কখনো না	১।মাঝেমাঝে	২।সপ্তাহে ১-৪ দিন	৩।প্রায় প্রতিদিন

৪৫। আপনি কি জন্ম নিয়ন্ত্রন বড়ি ব্যবহার করেন?

০ ।কখনো না	১।মাঝেমাঝে	২।সপ্তাহে ১-৪ দিন	৩।প্রায় প্রতিদিন

৪৬। নিয়মিত শারিরীক পরিশ্রম করেন কি না?

০। কখনো না	১। প্রতি মাসে ১-৩ দিন	২। সপ্তাহে ১-২ দিন	৩। সপ্তাহে ৩-৪ দিন	৪ ।প্রায় প্রতিদিন

৪৭। দৈনিক কত ঘন্টা ঘুমান? [ ] ঘন্টা

রোগ লক্ষণ ও সময়কাল/ রোগীদের প্রথম ক্লিনিকাল লক্ষণ

৪৮। আপনার নিম্নলিখিত লক্ষণগুলি আছে কিনা দয়া করে লক্ষ্য করুন

লক্ষণ	০। না	১। হ্যাঁ	হ্যাঁ হলে সময়কাল
ডান হাইপোকন্ড্রিয়াক / এপিগাস্ট্রিক ব্যথা			
পেটে ব্যথা			
ওজন হ্রাস			
দুর্বলতা			
পেট ফুলে যওয়া			
হলুদাভ চোখ এবং ত্বক			

জ্বর			
মাথা ব্যথা			
খুদা মন্দা			
বমি বমি ভাব			
শরীরের কোথাও বাদামি স্পট			
ডান কাধে ব্যথা			
অবসাদ			
চুলকানি			
মাঝেমাঝে অজ্ঞান হয়ে যাওয়া			
সহজে রক্তক্ষরণ হওয়ার প্রবণতা			
অন্যান্য............			

অতীতের চিকিৎসা সংক্রান্ত তথ্যাদি

৪৯। কখনো HBV সংক্রামণ হয়েছে কি না? ১।☐হ্যাঁ ০। ☐ না ; হ্যাঁ হলে সময়কালঃ

চিকিৎসাঃ ১।☐ হ্যাঁ ০। ☐ না ; হ্যাঁ হলে সময়কালঃ

ড্রাগের নামঃ ডোজঃ

চিকিৎসা বিরতিঃ ১। ☐হ্যাঁ ০। ☐ না ; হ্যাঁ হলে সময়কালঃ

৫০। কখনো HCV সংক্রামণ হয়েছে কি না? ১। ☐হ্যাঁ ০।☐ না ; হ্যাঁ হলে সময়কালঃ

চিকিৎসাঃ ১। ☐ হ্যাঁ ০।☐ না ; হ্যাঁ হলে সময়কালঃ

ড্রাগের নামঃ ডোজঃ

চিকিৎসা বিরতিঃ ১। ☐হ্যাঁ ০। ☐ না ; হ্যাঁ হলে সময়কালঃ

৫১। আপনার নিম্নের কোন রোগ আছে কিনা?

রোগের নাম	০।না	১।হ্যাঁ	হ্যাঁ হলে সময়কাল
Liver Cirrhosis			
Hemochromatosis			
NAFLD(Non-alcoholic Fatty Liver Disease			
Diabetes			
Stroke			
Hypertension			
Myocardial infection			
Asthma			
Choronic bronchitis			
Allergy			
Kidney disease			
Peptic gastric			
Biliary stone			
others................................			

৫২। আপনার লিভার ক্যান্সার ব্যতিত অন্য কোন ক্যান্সার ধরা পড়েছে কিনা? | ০। না | ১। হ্যাঁ |

৫৩। কখনো কোনো অপারেশন হয়েছেন কিনা? | ০। না | ১। হ্যাঁ |

যদি হ্যাঁ হয় তবে কোন ধরণের -

☐সাধারন অপারেশন ☐সিজারিয়ান অপারেশন

☐ডেন্টাল অপারেশন ☐অঙ্গপ্রতিস্থাপন অপারেশন

৫৪। কখনো রক্ত নিয়েছেন কিনা? | ০। না | ১। হ্যাঁ |

৫৫। কখনো ব্যবহৃত সূচ ব্যবহার করেছেন কিনা?

০। না	১। হ্যাঁ

৫৬। রোগীর বিএমআই = ওজন (কেজি) / উচ্চতা (মি^{২})

- ☐ স্বাভাবিক ওজন (বিএমআই ১৮.৫-২৪.৯৯)
- ☐ বাড়তি ওজন (বিএমআই ২৫-২৯.৯৯)
- ☐ স্থূলতা প্রথম পর্যায় (বিএমআই ৩০-৩৪)
- ☐ স্থূলতা দ্বিতীয় পর্যায় (বিএমআই ৩৫-৩৯.৯৯)
- ☐ স্থূলতা তৃতীয় পর্যায় (বিএমআই ৪০+)

স্থূলতা**?**

০। না	১। হ্যাঁ

লিভার ক্যান্সার সম্পর্কিত তথ্যাদি

৫৭। কোন ধরনের লিভার ক্যান্সারঃ ☐ প্রাইমারি ☐ সেকেন্ডারি

৫৮। কোন সময়ে লিভার ক্যান্সার ধরা পড়েছে? ☐

৫৯।. পূর্বে কখনো লিভার ক্যান্সারের নাম শুনেছিলেন?

০। না	১। হ্যাঁ

৬০। বিসিএলসি (BCLC) অনুসারে ক্যান্সার কোন স্টেজ এ আছে?

স্টেজ এ
স্টেজ বি
স্টেজ সি
স্টেজ ডি

৬১। কবে থেকে চিকিৎসা নেওয়া শুরু করেছেন? [ ]

৬২। কোন স্টেজ এ ধরা পড়েছে? [ ]

৬৩। নিম্নলিখিত কোন চিকিৎসা পদ্ধতির অধীনে আছেন?

সার্জারি	কেমোথেরাপি	রেডিয়েশন থেরাপি	ওষুধ গ্রহণ	অন্যান্য…………

৬৪। ক্যান্সার সনাক্তকরণে নিম্নের কোন পরীক্ষাগুলো করিয়েছেন?

সিটি স্ক্যান	এমআরআই	আল্ট্রাসাউন্ড	বায়োপসি	বায়োক্যামিকেল টেস্ট	অন্যান্য….

Tumor markers

65. AFP (ng/ml) : ☐ <200 ☐ 200-1000 ☐ >1000

66. CEA (ng/ml) :

67. Endoscopy of upper GIT :

68. The diameter of the tumor:

Number of tumors:

Physical examination

69. Anemia: ☐ Present ☐ Absent; If present, ☐ Mild ☐ Moderate ☐ Severe

70. Jaundice: ☐ Present ☐ Absent; If present, ☐ Mild ☐ Moderate ☐ Severe

71. Temperature (^{0}F) :

72.Respiratory rate (/min):

73.Pulse(/min):

74.Systolic BP (mm of Hg):

75.Diastolic BP (mm of Hg):

76.Hepatomegaly: ☐ Present ☐ Absent

77.Hepatic bruit:

78. Ascites: ☐ Present ☐ Absent; If present, ☐ Grade-1 ☐ Grade-2 ☐ Grade-3

79. Splenomegaly: ☐ Present ☐ Absent

80.Cachexia/ muscle wasting: ☐ Present ☐ Absent

81. Stigmata of CLD:

- ☐ Leuconychia
- ☐ Palmar erythema
- ☐ Spider angioma
- ☐ Gynaecomastia
- ☐ Testicular atrophy

82.Other systemic examination:

i) CVS:

ii) Respiratory:

iii) Nervous system:

iv) Locomotor:

Investigation reports

83. Hb% (g/dl) :
84. TC of WBC (....x 10^9):
85. Differential count- Neutrophil (%):
86. Lymphocyte (%):
87. Monocyte (%):
88. Eosinophil (%):
89. Basophil (%):
90. ESR(mm in 1st hour) :
91. Platelet count (......x10^{12}):
92. HBs Ag: ☐ Positive ☐ Negative
93. Anti HCV: ☐ Positive ☐ Negative
94. Anti HBc(T): ☐ Positive ☐ Negative
95. ALT (U/L) :
96. Prothrombin Time(sec):
97. INR:
98. Serum Albumin(gm/dl):
99. Serum Bilirubin(mg/dl):
100. Child-Pugh Score: ☐ Stage -A ☐ Stage-B ☐ Stage-C
101. Urine R/M/E :
102. Blood Glucose(RBS)(mmol/l) :
103. Serum Creatinine(mg/dl):

Imaging

104. Chest X-ray P/A view :

105. USG of the whole abdomen :

106. Triphasic CT/MRI :

Number of sol: 1 ☐ 2-3☐ >3☐

Size(cm) : ☐ <2 ☐ >2

Hepatic Segments involved:

Vascularity: ☐ Hypervascular ☐ Hypovascular

Portal vein invasion: ☐ Yes ☐ No

Extrahepatic metastasis: ☐ Yes ☐ No

107. **Cytopathology:**

108. **Medicine prescribed:**

Signature of Data Collector
